# Supplementary material for: A tool for measuring mental workload during prosthesis use: The Prosthesis Task Load Index (PROS-TLX)
Source: PLoS One. 2023 May 4;18(5):e0285382. doi: 10.1371/journal.pone.0285382 (PMC10159192; doi:10.1371/journal.pone.0285382)
Supplement: Attachment — Submitted filename: PLOS Reviewer Response.docx [file pone.0285382.s001.docx]

We would like to thank the reviewers for taking the time and effort to provide helpful comments on our work. You will see below that we have now responded to each of your comments (highlighted in red) in a point-by-point manner and made associated changes to the original manuscript. We hope we have now satisfied your initial concerns and would like to thank you for improving the strength of our submission.

Reviewer #1: This study outlines the development and validation of a way of measuring workload specific to prosthetic use. Technically, the methods are sound. However, there are some concerns.

First, in the survey of prosthetic limb users, there are some large limitations in the sampling. I understand that the sample size is similar to previous research, but you should still commend on the small sample size for determining a validated outcome measure and how representative it is of the wide population.

RESPONSE: Thanks for this comment. Although only a small number of prosthesis users responded to our survey it must be remembered that these responders were only responsible for confirming the relevance of the constructs that had been taken from a wide base of peer-reviewed literature on this topic that incorporated a much greater number of prosthesis users. This second, supplementary step, of having these constructs confirmed by current prosthesis users was not actually necessary for the development of the PROS-TLX such is the breadth of these workload demands reported in the literature but was important for understanding and identifying workload constructs that change with increased prosthesis control. We did this to add an extra layer of support to the design of the inventory (as per Wilson et al.). We agree that if we had just surveyed 8 prosthesis users and then designed the TLX on the basis of their responses, then this would have been a significant limitation but this was not the case - rather the 8 prosthesis users confirmed the relevance of constructs taken from a wider literature base. However, if the concern is that we have not captured an important construct, we would be happy for this to be highlighted.

It seems to be skewed to include a disproportionally high number of myoelectric users over body-powered device users, unless you are aiming for it just to be representative of myoelectric users. Also, please specify what level of amputations your participants had – if your sample includes both individuals with transradial and transhumeral amputations, the workload, their responses to the questions, and the reduction in the responses over time may be vastly different. It is likely not appropriate to combine them.

RESPONSE: The aim of this survey was to get expert insight into the relevance of different factors and see how they changed with increased prosthesis control, rather than to try to compare different populations and their experience over time.

We agree that the experiences of body-powered and myoelectric users would be different. In fact there are comments from one participant who had used both a myoelectric and a body powered prosthesis which explains why the experience is different. However, provided that the scale captures the major factors which describe workload pressures during prosthesis use, it does not matter that some factors will be more relevant to some users than others. The alternative is to develop specific scales for different populations, different prostheses etc. which is perhaps something that could be considered as a future step - although we doubt this would be desirable.

We also want to point out that this comment relates mainly to the magnitude of the workload experienced by prosthesis users, whereas our main reason for designing the PROS-TLX was to provide insight into the multidimensional nature of this workload in relation to constructs that are reported in the literature to be important for prosthesis users. In fact, we would go as far as to say that the magnitude and nature of the workload experienced by clinical users with different levels of amputation and different devices is likely to be highly individual. It is the PROS-TLX constructs that are representative of the demands that most users face (both able-bodied simulator users and clinical prosthesis users), rather than the magnitude of workload, as evidenced by a substantial amount of published research from which the constructs were taken. This distinction is paramount in understanding the design and utility of the study and the value of the PROS-TLX.

In addition, it is even more concerning that most of the questions are about first learning to use your hand, however, the respondents had a mean of 15 years wearing a prosthesis, which is far removed from first learning to use it. I do not think this this sample is appropriate for what you are asking.

RESPONSE: This is an interesting point, but we would argue that only prosthesis users with extensive experience are in a position to provide insights on their experiences of workload at the initial and latter stages of prosthesis control. It is possible that some respondents couldn't recall their initial learning situations although there is no indication in any comments that was the case (i.e., no participants stated they found it difficult or could not remember such situations). In contrast, several of the the qualitative answers provided detailed episodic accounts of their experiences, suggesting they were able to draw on their own experience to answer the questions.

The goal of the study was to understand, from the experience of prosthesis users, what factors contributed to experienced workload when first learning to use the hand, and also how each of these factors changed over time - it would not have been possible to establish this with a sample comprising only those first learning to use a prosthesis. For example, on page (7) we state in the methods:

“In addition to these questions, participants were asked the extent to which each construct was reduced as they became more proficient at using their prosthesis. They chose from six response options from ‘Not at all’, ‘A little’, ‘A moderate amount’, ‘A lot’ ‘A great deal’ or ‘I don’t know”. This question was asked as we were interested in the indices of workload that were specifically related to increased user proficiency and which are likely to reduce during training and rehabilitation…”.

It is well documented that mental workload decreases as patients become more skilled so we were interested in how each of these constructs may change over time. Some did not change or were unrelated to expertise (i.e., social stress) and so were taken out of the final TLX. Also, the point here of using the prosthesis simulator is important. People using this simulator for the first time are in an ideal position to comment on the workload they have just experienced without limitations of memory recall - this is an advantage of using such a device.

However, we agree that we could have been more explicit in outlining our goals of focusing on those constructs which have some relation to the proficiency of prosthesis control, and therefore display meaningful changes over time (i.e., when first learning vs after extensive practice). We have strived to make some of these points clearer in the aims section of the introduction (P5, L12).

Regarding the survey questions to this group, can you please provide some more information. 1) Please explain why you grouped concentration and mental fatigue together. 2) Please explain how much each of the domains decreased. You stated that mental demands decreased 100%, but by how much? 1 point on the Likert scale? 4 points?

RESPONSE: From a psychological perspective, the longer or harder you concentrate the more you will ultimately experience mental fatigue, so while they are different constructs they are highly related. We included concentration in this construct description to give added context to the online respondents. It is also worth noting that the final PROS-TLX contains the previously validated question “How mentally fatiguing was using your prosthesis during that task?” that is present in all previous validated, task-specific, TLX inventories (Harris et al., Wilson et al.,).

These percentages relate to the number of participants who agreed that these constructs are in some way important. We agree that this could have been explained more clearly in our original submission and we have now attempted to clarify this within the manuscript. Thank you for highlighting this.

We now explain the response option much more clearly:-

“Participants were asked the following questions related to each construct and were asked to respond using one of five options: ‘strongly disagree’, ‘disagree’, ‘neither agree or disagree’, ‘agree’, or ‘‘strongly agree’:-” (P6, L16)

The reported responses now reads:-

“Responses suggested that prosthesis users agreed or strongly agreed that Mental Demands (87.5%), Physical Demands (100%), Visual Demands (100%), Conscious Attention (87.5%), Frustration (100%), Situational Stress (50%), Social Pressure (87.5%), and Time Pressure (75%), were important constructs experienced when learning to use their prosthesis” (P, L)

The same changes have been made to the responses relating to how these demands reduce over time in the section that follows (P8, L1).

Regarding the development of the measure itself, I can see it’s usefulness and it seems to follow a logical development.

RESPONSE: Thank you for these positive comments, we are happy you see the utility in this measure.

I am unable to comment in detail on the suitability of the statistics.

My second concern is on the appropriateness of the discussion and conclusion. The limitation of validating a prosthesis workload questionnaire on able bodied participants is that the workload of using your anatomical hand is very low and the workload of using a brand new prosthesis device is very high. Saying that the PROS-TLX is sensitive enough to measure the different is not a strong statement as one would expect these two things to be extraordinarily different.

RESPONSE: We thank the reviewer for this comment but this is fundamental to the design of the study and is similar to the experimental protocol used to validate other task-specific TLX workload measures (Harris et al). We tested the sensitivity of the PROS-TLX in three conditions; 1) anatomical hand, 2) prosthetic hand and 3) prosthetic hand with pressure. The reason for adopting this experimental procedure was to assess specifically the sensitivity of the PROS-TLX across a range of conditions that differed in the magnitude and nature of workload experienced across different TLX constructs. We have clearly shown that the TLX is sensitive enough to capture these increased demands across these conditions.

We agree that it is somewhat obvious that workload across anatomic and prosthetic hand will naturally be extraordinarily different. However, the sensitivity of our scale is reflected in the significant correlations we have identified between behavioural and perceptual outcomes in first-time users of a prosthetic hand. For example, our correlations show that better performance with our device (i.e., greater proficiency) is associated with decreases across a multitude of workload constructs. This would suggest that our scale may be sensitive to understanding differences among prosthesis users who do or do not struggle at the early stages of learning.

We also want to point out that this comment again relates mainly to the overall magnitude of the workload experienced, rather than the multidimensional nature of this workload in relation to constructs known to be important for prosthesis users. The only aim of this experimental section was to show that responses in PROS-TLX measures change with associated increases in workload. It was not an aim of this study to show that the magnitude of workload reported by our able-bodied participants was representative of clinical users or users of other prosthesis devices, and nor do we suggest this. In fact, we would go as far as to say that the magnitude and nature of the workload experienced by clinical users with different levels of amputation and different devices is likely to be highly individual too. It is the PROS-TLX constructs that are representative of the demands that most users face (both able-bodied simulator users and clinical prosthesis users), rather than the magnitude of workload, as evidenced by a substantial amount of published research. This distinction is paramount in understanding the design and utility of the study and the value of the PROS-TLX.

If you want to comment on the sensitivity of the measure, I suggest measuring two more similar situations (between myo and body powered, between a basic myo hand and a multiarticulated one, etc.). It is also weak to use a lower extremity example in stating the clinical application of this measure for a prothesis user and imprudent to say you are confident that it is representative of lower extremity mental workload too. The entire paper is focused on upper extremity and there is no mention of any lower extremity application until the takeaway message.

RESPONSE: Please see the response above for our response to the first part of this comment and specifically to the distinction made between the magnitude and nature of the workload experienced. The constructs included in the TLX are based on known workload demands reported in both upper and lower limb studies so they are likely representative to users of both devices. We have shown that the TLX is sensitive enough to measure changes in these constructs experimentally. We are not saying that the magnitude of workload responses are similar across clinical users and simulator uses.

To say that the paper is entirely based on the upper extremity is a little unfair in our view. The introduction, the design of the PROS-TLX, and the discussion includes literature showing the perspectives of both upper and lower limb prosthesis users and how the findings might apply to each type of user. It is only the validation/experimental section that is entirely focused on the upper-limb. We concede that more work is needed using this inventory and from this review process alone we have had suggestions of further studies with clinical patients, those with different types of tasks, patients with different levels of amputation and different types of prosthesis. We are sure the reviewers agree that there is only so much we can do in one manuscript to show the utility of this inventory, but we believe this manuscript is the first step in this process and encourage others to explore its utility in other contexts.

However, to address this issue we have made considerable changes to our limitations section:

“We acknowledge that our survey respondents were primarily myoelectric upper limb users. It could therefore be argued that users with different levels of amputation or users of lower limb prostheses may have different experiences with each construct of workload over time. While this may be the case, the central constructs of the PROS-TLX were based on recurring themes across literature taken from both lower [6,19] and upper limb prosthesis users [18]. So while the individual importance of each workload construct may differ across users of different devices or levels of amputation, we believe that the importance of these constructs should still be relevant to prosthesis use in general. We are confident that they are more relevant than the most widely used measure for quantifying workload in prosthesis users, the NASA-TLX, which was designed for pilots during space flight.” (P22, L1)

Also, it is true – you have validated this measure in able-bodied participants and it is not representative of the clinical population. One might agree that it is relevant in the first 10 minutes of using a prosthesis for the first time, but after that it becomes irrelevant. This is a huge limitation and should be discussed as such. In many studies, I see the protocol piloted on able-bodied participants and then more thoroughly evaluated on people with amputations. I would strongly recommend evaluating this measure using actual prosthesis users.

RESPONSE: We tackle the issue of using a prosthetic simulator and highlight this as a potential limitation. We stated that:-

“It could also be argued that we have validated this workload measure in able-bodied participants and that the results might not reflect the workload experienced in the clinical population. While this may be true, research indicates that amputees and non-amputee users of a simulator report similar levels of workload on the NASA-TLX [28]. Also, previous research using prosthesis simulators has shown comparable kinematic profiles [29] visuomotor behaviours [3,6] and perceptual experiences [30] to prosthesis users, suggesting that using a simulator provides a useful surrogate to provide an insight into the sensory-motor deficits that prosthesis users face when learning and using a prosthesis [31].” In fact, using a simulator in this study could be considered as a strength as we have participants using a simulator for the very first time, so they are in an ideal position to comment on the workload they have just experienced without limitations of memory recall.

These references provide evidence that the way able-bodied participants move when using a prosthesis, the way they use their vision and the way they perceive objects are very similar. This similarity is probably amplified in our study where the mode of control (i.e., myoelectric) is identical to the mode of control used in the clinical population. Furthermore, a recent review [31 in our manuscript] has highlighted the utility, and challenges, of using simulators for understanding some of the psychological aspects behind prosthesis control and learning. Taking in consideration the distinction between the magnitude and nature of workload experienced (i.e., the experimental section was only designed to show that the TLX was sensitive enough to measure changes in different workload constructs) differences between simulator users and clinical users would be unlikely to have an impact on the results presented here.

Based on the difficulties (and uncertainty) in using prostheses and the similarities between simulator users and clinical users evidenced above, it is highly unlikely that it only applies for the first 10 minutes.

My only other comments would be about some questions in your abstract and introduction. Please clarify.

1) Does it requires emotional effort to use a prosthesis? 2) Why is using a prosthetic limb difficult? 3) Why is it clinically relevant to understand prosthesis workload? 4) Has the NASA-TLX ever been validates for use with prosthesis users?

RESPONSE: It is unclear whether emotional effort is required. We have simply indicated (based on previous research) that using a prosthesis is emotionally demanding.

Where we mention that using a prosthetic limb is difficult, in the next sentence we explain that “The absence of somatosensory feedback, impairments to motor control and problems with prosthesis fit can elevate levels of mental and physical effort, which have been linked to increased device dissatisfaction and high device rejection rates”. The link between workload and device dissatisfaction and rejection is why it's clinically relevant. Measuring the magnitude of workload is relatively easy but understanding the sources of workload that contribute to this overall magnitude is what is most relevant for understanding the nature (not magnitude) of the workload experienced. There is currently no tool that is designed for this purpose.

The NASA-TLX has never been validated for prosthesis use, we have now mentioned this. Despite this, it's the most commonly used self-report inventory used in prosthesis research of both lower and upper-limb and with individuals using simulators and clinician patients (REF). The NASA-TLX is only validated for the context of space flight and does not contain constructs that we know are critical sources of workload in prosthesis users. This is the rationale for this paper and despite possible criticisms of the PROS-TLX, we argue that it still represents a more appropriate measure than the NASA-TLX commonly used in this field.

You did a great job explaining different ways of measuring workload, their limitations, and why they are not clinically useful. I see the need for this measure and think it will be useful. I am concerned that it was formed and validated on a population that is not transferrable to the clinical environment.

RESPONSE: Thank you for these positive comments, we are happy you see the utility in this measure. We hope that our responses to comments related to the use of a simulator, together with the published research validating and advocating its value in research in this area have somewhat alleviated these concerns.

Reviewer #2: Major:

Strengths:

• The manuscript is well written and easy to follow.

• The need for the work is well justified.

• The potential significance is high as this work aims address a need to develop and validate a self-report measure of mental workload specific to prosthesis use.

• The approach taken mirrored the development of methods used in two other areas for modification of the TLX.

• Surveys and responses available on the Open Science Framework make the data and analysis available to other researchers.

• It was good to eliminate non-applicable constructs (e.g., social stress) from the measure based on the user responses.

• Exploring multiple metrics (e.g., functional task, eye tracking, etc.) improve the significance of the work.

• Convergent and divergent validity were appropriately established through correlation analyses with RSME and IMI, respectively.

RESPONSE: Thank you for highlighting the many strengths of our work.

Weaknesses:

• No persons with limb absence were used in the validation of the metric. Therefore, the conclusion that “the PROS-TLX captures the multidimensional nature of the cognitive, physical and emotional workload experienced by prosthesis users and provides……” This is the major reservation of this author to allow it for publication.

RESPONSE: The sample included were all prosthesis users albeit able-bodied but we take your point here and have softened these conclusions in the manuscript. We tackle the issue of using a prosthetic simulator and highlight this as a potential limitation. We stated that:-

“It could also be argued that we have validated this workload measure in able-bodied participants and that the results might not reflect the workload experienced in the clinical population. While this may be true, research indicates that amputees and non-amputee users of a simulator report similar levels of workload on the NASA-TLX [28]. Also, previous research using prosthesis simulators has shown comparable kinematic profiles [29] visuomotor behaviours [3,6] and perceptual experiences [30] to prosthesis users, suggesting that using a simulator provides a useful surrogate to provide an insight into the sensory-motor deficits that prosthesis users face when learning and using a prosthesis [31].” In fact, using a simulator in this study could be considered as a strength as we have participants using a simulator for the very first time, so they are in an ideal position to comment on the workload they have just experienced without limitations of memory recall. (P22, 21)

These references provide evidence that the way able-bodied participants move when using a prosthesis, the way they use their vision and the way they perceive objects are very similar. This similarity is probably amplified in our study where the mode of control (i.e., myoelectric) is identical to the mode of control used in the clinical population. Furthermore, a recent review [31 in our manuscript] has highlighted the utility, and challenges, of using simulators for understanding some of the psychological aspects behind prosthesis control and learning. Taking in consideration the distinction between the magnitude and nature of workload experienced - the experimental section was only designed to show that the TLX was sensitive enough to measure changes in different workload constructs. We are unsure of how any differences between simulator users and clinical users would have an impact on the results presented here.

In fact, using a simulator in this study could be considered as a strength as we have participants using a simulator for the very first time, so they are in an ideal position to comment on the workload they have just experienced without limitations of memory recall.

The validation portion of the study did not use any amputees and therefore the application to the amputee population (which is the point of the work) remains in doubt. The study that is referenced to show equivalence between able-bodied and amputee subjects [28] was done using EEG to control the device, which only tangentially appliable. At a minimum, if the validation testing was completed on a subset of amputees and shown to have similar results to able-bodied subjects, may be acceptable.

RESPONSE:

Please see our response above for the issue about using a prosthesis simulator.

It is important to also recognise that the validation of our scale was centred on whether our constructs of workload show some association with prosthesis proficiency and performance. In other words, we have shown that those who are better at using a prosthesis for the first time report lower levels of workload across a wide-range of constructs. Given that long-term prosthesis users experience workload issues related to our constructs, they are clearly relevant (to some extent) to amputee subjects. We hope that our scale will enable researchers to determine how each of these constructs differentially relate to prosthesis proficiency in amputee subjects. This is something we perceive as a strength of our scale that will lead to interesting comparisons between able-bodied and amputee individuals.

The study that is referenced did not use EEG to control a prosthesis; they used the same prosthesis and task setup as used in this study and measured cognitive effort through EEG, so this reference is entirely appropriate to support this point.

It is also important to note that your comment is related primarily to the magnitude of the workload experienced whereas the main reason for designing the PROS-TLX was to provide insight into the multidimensional nature of this workload in relation to constructs that we know (from research) are important for prosthesis users. It is the PROS-TLX constructs that are representative of the demands that most users face (both able-bodied simulator users and clinical prosthesis users), rather than the magnitude of workload, as evidenced by a substantial amount of published research. This distinction is paramount in understanding the design and utility of the study and the value of the PROS-TLX.

• The literature referenced to document the issues experienced by prosthesis users includes only two references and therefore is incomplete.

RESPONSE: This was an error (pointed out by the previous reviewer) that has been rectified.

• While the number of users (8) that were used to confirm the workload dimensions was justified by being similar to a prior study. Just because someone else used that number does not mean that it is an acceptable number and further justification is necessary.

RESPONSE: We are aware of this of course, but it must be remembered that these responders are only responsible for confirming the constructs that have been consistently reported in the literature as valid sources of cognitive, physical and emotional demand. The synthesis of this literature will be based on a much greater number of prosthesis users throughout the published literature. The supplementary step, of having these confirmed by current prosthesis users was not actually necessary for the development of the PROS-TLX such is the breadth of these workload demands reported in the literature. We did this to add an extra layer of support to the design of the inventory (as Wilson et al) and to identify workload constructs that reduce over time in relation to increases in prosthesis control. If we had just surveyed 8 prosthesis users and then designed the TLX on the basis of their responses, then we agree that this would have been a significant limitation - we did not do this. However, if the insinuation is that we have not captured an important construct, we would be happy for this to be highlighted.

• Of the 8 individuals that were used, 7 of the 8 were myoelectric users and therefore the results are likely cannot be generalized to the body-powered user population. This should be stated as a weakness.

RESPONSE: Please refer to the comments above that we believe has addressed this concern.

We agree that the experiences of body-powered and myoelectric users would be different. In fact there are comments from one participant who had used both a myoelectric and body powered prosthesis which explains why the experience is different. However, provided the scale captures the major factors which describe workload pressures during prosthesis use, it doesn't matter that some factors will be more relevant to some users than others. The alternative is to develop specific scales for different populations, different prostheses etc. which is perhaps something that could be considered as a future step - although we are unsure if that would be desirable.

We have now added a specific comment related to this issue in our limitations section:

“We acknowledge that our survey respondents were primarily myoelectric upper limb users. It could therefore be argued that users with different levels of amputation or users of lower limb prostheses may have different experiences with each construct of workload over time. While this may be the case, the central constructs of the PROS-TLX were based on recurring themes across literature taken from both lower [6,19] and upper limb prosthesis users [18]. So while the individual importance of each workload construct may differ across users of different devices or levels of amputation, we believe that the importance of these constructs should still be relevant to prosthesis use in general. We are confident that they are more relevant than the most widely used measure for quantifying workload in prosthesis users, the NASA-TLX, which was designed for pilots during space flight.”

• Limb users were surveyed to confirm the importance of constructs that were taken from literature and prior measures, but were not engaged to include/develop constructs that were likely not have captured through these other mechanisms.

RESPONSE: Prosthesis users were free to suggest other constructs of interest (see their responses that are available in the links to the OSF) and we even picked out the construct of ‘uncertainty’ because of this exact process. This was then added to the PROS-TLX. We explained this on page 8.

• There is a large concern that the validation study used only the coin task of the SHAP with a multi-articulating prosthetic hand. Multi-articulating hands are particularly poor at picking up small objects (as compared to gripper terminal devices or body powered prostheses) and therefore this is a highly frustrating task that is not representative of most of the tasks that are done with a prosthesis. This is especially true for unilateral amputees that will likely just complete the coin task with their intact hand. Other bi-manual tasks would be informative.

RESPONSE: We thank the reviewer for this comment. However, we purposefully picked a task that we knew was somewhat frustrating, placed high demands on visual attention and was mentally, physically and emotionally demanding. This was the primary aim of the manipulation: we needed to see if the PROS-TLX would measure changes in these variables and also how these changes related to more objective measures like gaze behaviour. This is a very typical approach taken by other studies from experimental psychology that have designed and validated task-specific TLX measures. (Harris et al., Wilson et al.,). The only purpose of this experiment was to examine if task-induced changes in the mental workload could be captured in changes in PROS-TLX responses, so we therefore chose a task suitable for this purpose.

• The SHAP is specifically designed as a timed test, not to be performed at a natural speed. How can you know if subjects where subjects were placing their priorities for completing the task (speed vs. drops) in the pressure condition? These are obviously conflicting demands. Also, if they were told not to rush during the non-pressure condition, should time be compared during non-pressure conditions?

RESPONSE: Similarly as above, this was just a psychological manipulation designed to induce stress and time pressure to see if the TLX would be sensitive to these emotions (it was). Yes, it is appropriate to compare time between pressure and no-pressure conditions as this serves the purpose of demonstrating that participants were actually abiding by the experimental manipulation. Studies that have designed and validated task-specific TLX measures have used very similar manipulations (Harris et al., Wilson et al.).

• Why were subjects asked to complete the Part 2 comparisons ‘from using the prosthesis in general rather than in any one condition’? The only condition they had used it in was the coins task and, since they are not prosthesis users, didn’t have any other usage to compare against. Also, these comparisons of weights do not appear to have been presented in the results.

RESPONSE: We thank the reviewer for this comment but would like to clarify some aspects of the study design. The participants used the prosthesis in two conditions: the prosthesis condition and the pressure condition. Part 2 of the TLX is the weighting aspect of the inventory. We wanted to weight the user's scores from ‘general’ prosthesis use and use this weighting to derive the scores from each of the pressure vs no pressure conditions. This is typical when designing and validating task specific TLX measures (see Harris et al.) and fundamental to the design of the original TLX. Weights by themselves are not that important and are only used to compute the overall workload score as per the original TLX and other task-specific TLX measures. We have added some of this detail to the manuscript to aid clarity.

• Since time pressure did not show a significant increase in workload scores, shouldn’t it be removed from the PROS-TLX?

RESPONSE: There was a significant increase in time pressure in the ‘pressure’ condition. Time pressure was also related to visual attention, stress and overall workload score. Due to the clinical importance of time in regards to functional assessments (e.g., SHAP), this construct is critical in many, but not all, situations a user may face.

• It is likely an overstatement to claim that the PROS-TLX is sensitive enough to measure workload characteristics beyond differences between intact hand and prosthesis use as there was no comparison between two prosthetic interventions that have different mental loads (e.g., 2-site myoelectric control of a hand and wrist vs. pattern recognition control of a hand and wrist).

RESPONSE: As above, there was a comparison between two prosthetic interventions that have different mental loads because we had a no pressure vs pressure condition where the prosthesis was used - this was critical to the experimental design of the study. These conditions did have different workloads as evidenced by the data in our paper. The PROS-TLX was sensitive enough to measure changes in workloads across these conditions.

• The applicability and generalizability to lower limb amputees needs to be removed from the document as no LL amputees or testing with lower limb prostheses was performed in this work. There are already concerns about generalizability to even body powered upper limb amputees, much less lower limb.

RESPONSE: We agree and although we believe, based on published literature, that these constructs are important for lower-limb users as well we have now outlined that support for such conclusions is needed in future research.

In the limitations we now state:

“We acknowledge that our survey respondents were primarily myoelectric upper limb users. It could therefore be argued that users with different levels of amputation or users of lower limb prostheses may have different experiences with each construct of workload over time. While this may be the case, the central constructs of the PROS-TLX were based on recurring themes across literature taken from both lower [6,19] and upper limb prosthesis users [18]. So while the individual importance of each workload construct may differ across users of different devices or levels of amputation, we believe that the importance of these constructs should still be relevant to prosthesis use in general. We are confident that they are more relevant than the most widely used measure for quantifying workload in prosthesis users, the NASA-TLX, which was designed for pilots during space flight.” (P22, L1)

Minor:

Weaknesses:

• The Data Availability Statement does not describe where the data can be found.

RESPONSE: Links to the data are found throughout the whole manuscript, we have now added this link to the availability statement. Thank you.

• The data (at least for the coin drops in Figure 2B) does not appear normally distributed and is identified in the text as non-parametric. Therefore, box plots are a more appropriate way to visualize the data (and not the dot and whisker that is used).

RESPONSE: For visualisation purposes we have included all data points using a jitter plot placed behind our measures of central tendency and variance. The use of jitter points allows the visualisation of data distribution above and beyond what can be presented using box and whiskers. We believe box and whiskers would look very clunky in these graphs given how many are placed next to one another and would much prefer to keep the visualisation the same.

• It is unclear why the questions that were asked of the amputee users (Page 12-13) ask about ‘first learning,’ ‘learning to use,’ and ‘learning to move.’ Why were different sentence structures used?

RESPONSE: We previously explained that ‘This question was asked as we were interested in the indices of workload that were specifically related to increased user proficiency and which are likely to reduce during training and rehabilitation”. Workload is higher when first learning to use a prosthesis and research shows these demands are reduced with increased proficiency. This was just how we phrased these sentences to make them as clear as possible.

We have revised our aim section in the hope of further clarification:-

“In this paper we aimed to develop and validate a self-report measure specific to the multidimensional nature of the workload experienced during prosthesis use - The Prosthesis Task Load Index (PROS-TLX). By adopting methodologies from previous research that have designed and validated task-specific workload measures for surgery (SURG-TLX) [15], driving [16], and simulated (virtual) environments (SIM-TLX) [17], we first identified constructs of workload from current literature and prosthesis user feedback. From this, we targeted those constructs related to the proficiency of prosthesis control which will be more meaningful to rehabilitation and technological advancements. Next, we sought to validate this inventory experimentally by manipulating sources of potential workload during upper-limb prosthesis use in abled-bodied participants using a prosthetic hand simulator. This enabled us to examine how experimental conditions differing in workload demands were reflected in changes in the constructs of the PROS-TLX.” Page 5.

• Grouping various constructs under item 5 appears inappropriate. ‘Insecure,’ ‘discouraged,’ ‘stressed,’ ‘irritated,’ and ‘annoyed’ are all very different constructs. (maybe you could claim that irritation and annoyance are similar enough). How are you able to know which of these verbs were the ones that users were focused on when answering the question?

RESPONSE: These are validated items taken from the original NASA-TLX and previously published studies have used the same wording in the design and validation of similar task-specific TLX measures.

• A similar comment could be made for items 6 & 7, but the difference between ‘anxious’ and ‘stressed,’ and ‘hurried’ or ‘rushed’ is less severe.

RESPONSE: Please see our response and published literature cited above.

• Please clarify on page 15 (of the PDF) that the hand is controlled by ‘isometric’ muscular contractions.

RESPONSE: This has been added.

• Please describe why ECR and FCR muscles were chosen as opposed to EDC, ECU, FCU, finger flexors, etc. The proximity to the brachioradialis that will be active supporting the weight of the simulated prosthesis could create crosstalk with the radialis muscles. Also, please describe in more detail the methods used to locate and identify these muscles.

RESPONSE: Muscles were located based on the touch of the researcher as the participant was contracting each muscle. Once identified each electrode was placed on the belly of each muscle. Activation of the extensors trigger the opening of the hand whereas activation of the flexors trigger the closing of the hand. Each participant was asked to open and close the hand until they could operate it when prompted on 10 consecutive attempts. They then had 2 coins to practice the task. This procedure minimised any cross talk with other muscles. This detail has now been added. (P10, L8)

• Page 17, describes ‘After completing each task,’ but there was only one task (coin task), correct? Please clarify.

RESPONSE: This should have read ‘condition’ not task. Thank you, this has been changed.

• Does the 20-point Likert scale match the NASA-TLX?

RESPONSE: Yes. we have now made this clear within the paper (P11, L15).

• Please provide more details on how the 28 pairwise comparisons between dimensions were administered in the text and results. I looked on the link provided on page 17 of the PDF but did not see them there and they are not discussed in the analysis.

RESPONSE: These instructions are outlined at the top of the Part 2 of the TLX (see OSF links) but we agree that this needs further clarification in the manuscript itself. We have now added this detail to the section in question.

“For this, each dimension was paired against each other and participants were asked to choose the construct that provided the most significant source of workload across each pair.” (P11, L18)

• Please provide a brief description of Conover comparisons are as they are not a commonly utilized metric as compared to other approaches and it is likely that readers (like this reviewer ) will be unfamiliar with them.

RESPONSE: The Conover test is another post-hoc test used after a significant Friedman test - it is also the default post hoc test for this analysis in JASP (the statistical software used in this study). The suitability of this test and the equations underpinning it can be evidenced here:- <https://www.real-statistics.com/anova-repeated-measures/friedman-test/friedman-test-post-hoc-analysis/>.

• The bottom of page 23 of the PDF states: Analysis of the relationships between PROS-TLX constructs showed moderate to strong correlations between almost all PROS-TLX dimensions and every dimension correlated with total workload score, as expected (see Figure 4). However, correlations b/t each of the PROS-TLX and total workload score are not presented here, they are only correlated to Performance time, coin drops and hand fixation rate. It may be that the captions and references to Figure 4 and Figure 5 are switched in some places. This would be true if the caption for figure 5 removed the reference to ‘total workload’ and replaced it with ‘hand fixation rate.’

RESPONSE: You are correct, we uploaded Figure 4 and 5 in the wrong order. Apologies, we have now changed this. Thanks for highlighting this error.

• In the discussion, can you clarify why, if the goal of the study is to design a self report measure of mental workload, why physical demands are included in the analysis. Is there evidence that these two are linked as they seem to be very different constructs?

RESPONSE: These constructs were included in the original NASA-TLX and in every other task-specific TLX measure designed and validated since. The constructs of mental and physical effort are linked and it is well evidenced that increased physical fatigue elicits concomitant increases in cognitive process implicated in mental fatigue (Gibson & Foster, 2007).

Gibson, A. S. C., & Foster, C. (2007). The role of self-talk in the awareness of physiological state and physical performance. *Sports Medicine, 37*(12), 1029-1044.

• Please provide a more detailed description of the main paragraph of page 26 of the PDF. The data from figure 4 shows that, while significant, the correlation between hand fixation rate and the visual demands question are only 0.2, vs. much higher correlations between other factors and measures.

RESPONSE: It is indeed interesting that the relationship between subjective and objective indices of visual demands were not stronger. We suggest this may be driven by the fact that eye trackers only capture the overt allocation of gaze and maybe participants were using covert attentional strategies (i.e., peripheral vision) to monitor hand location.

We have now added this comment to our this section:

“However, it is notable that the relationship between perceptions of visual demands and hand fixation rate was weak (r = .02). A potential reason for this may be due to the fact eye-trackers only capture the overt allocation of gaze and maybe participants were using covert attentional strategies (i.e., peripheral vision) to monitor hand location.” (P21, L4)

• References listed for background literature used in the development of the measure was listed as [5,18] earlier in the manuscript and then [6,19] and [18] in the discussion. Please clarify which is correct and make them consistent.

RESPONSE: Thanks for spotting this inconsistency. We originally meant to highlight the associated papers as examples “[e.g., 5,8]” but we have now highlighted the papers from the introduction that highlight any support for the PROS-TLX dimensions included. We have now changed this to :-

“First, we consulted the literature that has documented some of the issues reported by prosthesis users [2,3,4,5,6,7,10,13,18,19,20]”.

In the discussion this now reads:-

“While this may be the case, the central constructs of the PROS-TLX were based on recurring themes across literature taken from both lower [2,5,9,13,19] and upper limb prosthesis use [3,4,5,6,7,18,20]”.
