# Supplementary material for: A tool for measuring mental workload during prosthesis use: The Prosthesis Task Load Index (PROS-TLX)
Source: PLoS One. 2023 May 4;18(5):e0285382. doi: 10.1371/journal.pone.0285382 (PMC10159192; doi:10.1371/journal.pone.0285382)
Supplement: Attachment — Submitted filename: Reviewer Responses TLX R2.docx [file pone.0285382.s002.docx]

Reviewer #1: Thank you for responding to the comments. Your responses helped to clarify several things. However, I think there are still some weaknesses that need to be addressed.

- In the abstract, what is your rationale for saying that when using a prosthesis, a high level of emotional effort is experienced?

RESPONSE: The literature we reference in relation to this comment suggests that emotions like frustration and anxiety are often experienced by prosthesis users. This was referenced within the first parts of the introduction. We have not added any more detail in the abstract as it doesn’t seem appropriate, but we have added some further detail for clarification in the introduction. (P3, L4)

- Also, in the abstract, you need to include that this paper focused on upper extremity. While you have commented on its applicability to lower extremity in the future, this paper simple did not do that, and you need to provide this specific information to your readers. This is also true in the background. You need to explicitly state that you are hoping that this measure will be applicable to both upper and lower extremity prosthesis users, but in this study, you are specifically looking at upper extremity.

RESPONSE: We agree and have now done this here and throughout the paper.

- You are still missing basic demographic information about the individuals who completed your survey including level of amputation.

RESPONSE: Age, device type, sex and the number of years of experience using a device was all the demographic information we collected. In retrospect, level of amputation might have been interesting, however, in such a small sample of respondents its unlikely to have been informative as it would have been very difficult to compare between so few participants. We previously outlined in the limitations section that the level of amputation might elicit differences in workload and highlighted this has a future avenue for further research. These comments remain in the revised limitations section. (P22, L11)

- These are still large issues in your methods. You stated, “The goal of the study was to understand, from the experience of prosthesis users, what factors contributed to experienced workload when first learning to use the hand, and also how each of these factors changed over time”. You are asking questions that, while they are answering, are likely not accurate responses. I appreciate that you were using these individuals to confirm that the items you included were relevant. However, an average of 15 years is too long of a recall period to draw conclusions about learning to use a prosthesis. While no participate may have commented they could not remember, I challenge you to remember the detail of an experience you had 15 years ago. To address this, you need to include a comment on recall bias and address this length of time. There is lot of research on recall and memory. I suggest doing more background reading and commenting on this in depth.

RESPONSE: We have now added this as a limitation of the study.

- You mention how you removed the social stress section. Does the social stress of how they are perceived impact their workload and control because more of their brain is dedicated to dwelling on this aspect? This seems to be the closest thing to emotional demand that you mentioned earlier but now you removed it.

RESPONSE: As we mentioned in the previous response and in the previous revision of the paper, we wanted to focus on the sources of workload that change with increased prosthesis control. Many stable factors, like personality type for example, will impact on sources and magnitude of social stress as will things like the aesthetics of the device itself. Whilst it is possible that cognitive resources are devoted to dwelling on social stress, in terms of emotions we are principally talking about anxiety and frustration because these are more relevant to device proficiency than social stress.

We previously explained…

“The social stress construct was omitted from the final version as the free text comments revealed that this construct had little relevance to user proficiency and was more related to stress about the aesthetics of the device and others’ knowledge about the personal circumstances around their disability. Although these are obviously important psychosocial aspects of a user's experience, they were not deemed to be relevant to the context for which the PROS-TLX is primarily designed (i.e., research related to workload and prosthesis control).”

- Bringing in lower prosthesis users in the discussion is unnecessary and confusing to the reader (page 21, line 16). It is already concerning you are expanding the application to users with upper extremity prostheses, let along lower extremity ones.

RESPONSE: We agree and have now taken reference to lower limb prosthesis users out of the paper and just comment on this as a future research direction.

- There are still significant issues with talking about the validation of a measure without using individuals with upper extremity limb loss. Make this abundantly clear in the discussion and results. You have completed step 1, but it is not yet suitable for clinical use.

RESPONSE: We have now included a paragraph towards the end of the discussion in which we make this point clear and call for future research to validate the PROS-TLX measure in a clinical population. (P22, L11)

In addition, your results show difference between no prosthesis and a prosthesis but not between prosthesis conditions. The difference between using your hand and using a prosthesis is massive and a measure does not have to be very sensitive to pick this up. There is evidence that this measure is not sensitive enough to compare between conditions in a prosthesis. Therefore, it is likely an overstatement to say that it can be used to understand prosthesis control, test interventions, monitor the nature of the workload experienced and how it changed through rehabilitation, assess developing technologies, etc. While this work has importance, you need to be realistic about stating it’s importance and the further work needed.

RESPONSE: Apologies, but there might be a misrepresentation of the results of the study. The participants used the prosthesis in two conditions: the prosthesis condition (low mental effort) and the prosthesis + pressure condition (high mental effort). Total workload was different across each of these conditions in the manner we hypothesised. To add clarity, we have now renamed the experimental conditions to anatomic, prosthesis and prosthesis + pressure conditions. It should also be reinforced that our correlational analyses indicated several meaningful relationships between workload, performance, and visual attention. As such, those who reported higher PROS-TLX scores also performed worse and relied more on their vision (to name a few). This would indicate that this workload is sensitive to within-group variations in the workload experienced during prosthesis control.

**Reviewer #2:** The authors made changes to address several of the comments that were discussed by the reviewers that have improved the manuscript. They also made reasonable counterarguments in several cases (e.g., regarding sensitivity, potential applicability to non-myo users, justification for 8 subjects due to the goal of their involvement, etc.). However, the changes that were made have not reached the level to make this reviewer willing to suggest that the work is ready for publication.

Overall, there appeared to be a general reluctance by the authors to address the questions/concerns that were shared by both reviewers in the manuscript. In general, even if the authors are correct on a given point, if multiple reviewers have questions/concerns about a given topic, it requires a more detailed explanation and clarification in the manuscript. It is likely that many other readers of the manuscript will likely have less expertise than the reviewers in understanding the concept and therefore more clarification is justified.

RESPONSE: Apologies if we gave this impression. We strived to incorporate your comments where we felt they were justified, clarify aspects when needed and argue against the ones we felt were either misinterpreted or we simply didn’t agree with. We have now made further changes and we hope to have satisfied the reviewer this time around.

For the reasons described above, it is important to include changes in the manuscript to further explain and clarify questions brought up by the reviewers. The authors responses to many these comments was on point, and the manuscript would benefit from the inclusion of these explanations in the final draft. For this reviewer (#2) these include:

o Reasoning/logic behind picking the coin task for the SHAP and how, while the SHAP is typically timed, the authors chose to modify the approach.

RESPONSE: We selected the coin task as it is very challenging and therefore allows for a wide variation in performance/skill adaptation. A simple jar lifting task would be easier and therefore have a lower ceiling for difficulty and workload. We also chose this task because evidence from previous research indicates that this task places high demands on visual attention and is mentally, physically and emotionally demanding. As such, it was an appropriate task to use to use to validate many of the constructs assessed in the PROS-TLX. We have added this information on P10 L17-19.

o Stressing wording was taken directly from the original NASA-TLX related to insecure/stressed/irritated/stressed/hurried/rushed/etc.

RESPONSE: We have now stated explicitly that the wording of these items in the PROS-TLX were taken directly, or adapted from, the NASA-TLX (P9 L14).

o Description of Conover comparisons

RESPONSE: We agree that more detail was needed to describe these comparisons and have added this on P14 L12-13.

o Description of the inclusion of physical demands

RESPONSE: We have now clarified that we are referring to fatigue when taking above physical demand in the introduction and have also outlined that this dimension is from the original NASA-TLX.

o That the experimental session was only designed to show that the TLX was sensitive to measure changes in different workload constructs and not between different prosthetic interventions.

RESPONSE: We have now done this.

Comments related to this reviewer’s (#2) responses is below

Major:

The overall conclusions need to be further softened and the limitations of the study expanded. Every study has its limitations, that is completely acceptable, and the limitations do not all have to try to be justified away. I understand why the authors would prefer to not list out the limitations of the study, but they exist and should be described in detail. I believe that this manuscript is an important first step in the development of the instrument, and it is worthy of publication, but limitations need to be called out with greater emphasis.

RESPONSE: Thank you. We have further expanded our limitations section to include three paragraphs discussing each limitation in turn and contextualising them with evidence. For example, readers might not be aware of the experimental work using prosthesis simulators and may immediately dismiss our work due to the lack of knowledge of these findings. Including this context serves to educate the reader so that they can make an informed decision of the magnitude of the limitations we have outlined. P22, L11

For example, please add “the PROS-TLX captures the multidimensional nature of the cognitive, physical and emotional workload experienced by able-bodied prosthesis users using a prosthesis simulator and provides…” Following this with a description of how the able-bodied results may translate to amputee users would be appropriate.

RESPONSE: We have now done this. P23, L24

The authors were accurate to correct this reviewer that the study that is used to provide evidence that amputees and non-amputee users of a simulator report similar levels of workload on the NASA-TLX [28] did not use EEG for the control system. However, after further review, it was discovered that this study used only used a single subject (n =1), using a very advanced, implantable control system (and therefore would be argued to not be ‘entirely appropriate’ as stated by the authors). In addition, this study compared the single amputee’s results to two able-bodied subjects, and report that ‘the group was not large enough to draw any statistical conclusions.’ Therefore, equivalence between amputee and able-bodied users cannot be shown by this work. These limitations should be noted.

RESPONSE: We agree with your argument here and so have decided to delete reference to this study in the revised limitations section.

I think the authors make a good argument that simulators can be shown to be fairly representative of other aspects of actual prosthesis use. While I understand that there are references showing the relative equivalence between AB users using simulators and amputees, the study would undoubtedly be strengthened by performing testing with amputee subjects. I agree with the authors claims that the results are relevant to amputees, but they are not results obtained directly from the population that this metric will be applied to (i.e., amputee users) and this needs to be stressed as a limitation.

RESPONSE: We have now strived to do this more clearly. In the final paragraph of our limitations section (P23, L9-21) we outline that further research is needed using the PROS-TLX in clinical populations. We also conclude our paper by inviting researchers and applied practitioners to use the PROS-TLX with clinical patients to strengthen support for the use of this inventory (P24 L15-20).

Please clarify why using the simulator could be considered a strength. Wouldn’t an amputee user be able to have similar comments on the workload they just experienced? What are the limitations that are created by memory recall? Ideally new, amputee subjects would be used here to get the best of both worlds, right?

RESPONSE: We agree that testing with new, amputee subjects would be the best of both worlds but there are still benefits associated with using able-bodied participants using a simulator. For example, (i) it allowed us to test lots of users very quickly and (ii) it allowed us to take measurements from them on their first occasion using a device, which was logical for this initial validation. Furthermore, this initial validation with the simulator has proven critical in helping us get ‘buy-in’ from clinicians (we are psychologists) and we are currently working with them and upper-limb patients on further validation, but this takes time and would have been more difficult without the simulator-based evidence. However, we agree that it wasn’t appropriate to claim this as a strength within the paper and so we have deleted this comment in the revised limitations section.

Further results of the weights that were reported by subjects should be reported in detail. These data would provide useful evidence to other researchers to understand and interpret the work, and potentially apply in their own use.

RESPONSE: Weights by themselves are not that important and are only used to compute the overall workload score as per the original TLX and the other task-specific TLX measures outlined in the manuscript. For example, they would not show any difference between either the prosthesis vs. the prosthesis + pressure condition even though both significantly differed on total workload scores. However, we have uploaded these data to our OSF page so that they are now freely downloadable for any interested reader.

Unless the normality of the data presented (e.g., in figure 2b) can be shown to have a normal distribution, the presented method of visualization is incorrect and needs to be rectified. The authors state that a box and whiskers would ‘look very clunky.’ This reviewer would find either 1) analyzing and presenting results showing the data are normally distributed or, if not, 2) any way of visualizing the non-normal data that the authors deem visually pleasing, to be acceptable.

RESPONSE: We have changed these to boxplots as previously requested.

Minor:

Regarding muscle location finding:

• By ‘touch’ do you mean ‘palpation’?

RESPONSE: Yes. This was in our response not the manuscript so no change needed.

• Please remove the sentence, ‘This procedure minimized any cross talk with other muscles’ as this is unlikely to be true given the ECR/FCR proximity to the brachioradialis….frankly it is details like this that add to the reservations about using able-bodied subjects using a simulator and saying they are a suitable stand in for actual amputees using a prosthesis.

RESPONSE: We have now deleted this sentence as requested..

In attempting to align reference numbers it appears as thought Reference 9 and 10 do not agree between earlier in the manuscript and in the discussion.

RESPONSE: Thanks for spotting this. We have now rectified it.
