# Supplementary material for: A tool for measuring mental workload during prosthesis use: The Prosthesis Task Load Index (PROS-TLX)
Source: PLoS One. 2023 May 4;18(5):e0285382. doi: 10.1371/journal.pone.0285382 (PMC10159192; doi:10.1371/journal.pone.0285382)
Supplement: Attachment — Submitted filename: Reviewer comments and response_R3.docx [file pone.0285382.s003.docx]

Editor: Please could you make a decision on this manuscript without send this out for further review. These are very minor changes, R2 has suggested they don’t need to review this again and it has been over 12 months in revision to date. We have further studies waiting to be publish using the TLX and a fast decision would be most appreciated. Many thanks.

Many thanks for both reviewers for their comments. We have changed everything suggested here.

Reviewer #1: Thank you for addressing most of my previous comments. I still have a few outstanding comments that need to be addressed.

- In the abstract, I still disagree that a high level of emotional effort is experienced when using an upper limb prosthesis. While this may be true for some people, there are many prosthesis users who do not experience high emotional effort (or high physical or mental effort for that matter.). Many users are very comfortable using their prostheses. Please reduce the severity of the descriptor or reword this to be more encompassing of all users of prostheses.

RESPONSE: This has been changed.

- Also in the abstract, it is still my opinion that you remove “lower-limb amputees” from the further work section. This paper is focused on upper extremity. It is ok to include it in the limitations section of the discussion, but I do not think it belongs in the abstract. - Also, “upper-limb […] amputee users of prosthetic devices” is not good grammar.

RESPONSE: We have changed this now.

- Page 22 Line 15 “Primarily myoelectric upper limb prosthesis users”

RESPONSE: Thanks, we have added this.

- Page 23 lines 3-10 – And also more work is needed on individuals in rehab actually learning how to use a hand for the first time.

RESPONSE: We have added this.

Reviewer #2: Reviewer #2:

Overall, this round of edits was responsive to the primary concerns of this reviewer and, particularly the expansion of the description of the limitations, has substantially increased my level of enthusiasm for publication. With one final, minor edit, I believe this should be in good shape. If the editor is comfortable with the response by the authors to this item, this reviewer does not need to do any additional review on this paper.

• In response to Reviewer #1s comment on level of amputation, the authors claim that they addressed this issue on P22, L11, but I didn’t see it there (or elsewhere).

RESPONSE: In the previous revision we wrote ‘It could therefore be argued that users of different devices (e.g., body-powered) or with differing levels of amputation may have different experiences with each workload construct.”. This is now located on P22, L15
